# Epidemiology of human exposure to rabies in Nunavik: incidence, the role of dog bites and their context, and victim profiles

**DOI:** 10.1186/s12889-020-08606-8

**Published:** 2020-04-29

**Authors:** Sarah Mediouni, Mario Brisson, André Ravel

**Affiliations:** 1 Groupe de recherche en épidémiologie des zoonoses et santé publique, Faculté de médecine vétérinaire, Université de Montréal, Saint-Hyacinthe, Quebec Canada; 2 Ecole de santé publique, Université de Montréal, Montréal, Quebec Canada; 3 Direction de la santé publique du Nunavik, Kuujjuaq, Quebec Canada

**Keywords:** Animal, Dog, Injury, Bite, Children, Nunavik, Young adult, Rabies, Management, Public health

## Abstract

**Background:**

In Nunavik, Arctic rabies is still endemic due to a spillover from wildlife to dogs. The prevention of human exposure and the management of potential exposure is a significant public health concern in this region.

**Methods:**

This study retrospectively describes cases of potential exposure to rabies in humans as reported to the Nunavik Public Health Board through their registry of reported cases. We used multi-correspondence analysis as well as univariable and multivariable regression models to test for differences between children and adults in reported cases, and to examine the contexts of exposure to dogs and dog attacks.

**Results:**

From 2008 to 2017, 320 cases of potential exposure to rabies were reported, 92% of which were linked to dogs. The annual incidence rate was 2.5 per 1000 people. The incidence increased significantly during the study period, although the reasons for this are unclear. Fifteen cases of exposure were with rabid animals, mostly dogs (9 of 15). No human cases of rabies occurred thanks to adequate medical case management. Two specific profiles for potential exposure to rabies were identified based on age and gender. The first was children (< 15 y/o), male or female, who were more likely to be exposed through playing with dogs and were more often injured in the head and/or neck. The second was young male adults (aged 15 to 34 y/o), who were more involved with wildlife than other age groups and mostly injured in the upper limbs and as a result of a reaction by the animal.

**Conclusion:**

Rabies is a real public health threat in Nunavik. Potential human exposure needs to be prevented, and prevention measures should be tailored to the two risk profiles identified based on age, gender and animal species involved.

## Background

Dog attacks and bites have been acknowledged as a public health problem for decades [1, 2]. Among the various issues related to such injuries, rabies exposure remains the most serious, making it a major concern for health authorities [3–6]. In Canada, a study conducted in 22 municipalities showed an annual dog bite incidence rate ranging from 0 to 1 per 1000 people [7]. However, reported dog bites represent only a small fraction of the true incidence, which is between 20 to 50% [8–10]. In fact, studies conducted in the United States showed that annual dog bite incidence ranged from 1 to 18 per 1000 people when unreported bites are taken into account [11, 12].

In the province of Québec, Canada, a 2016 report by the Ministry of Agriculture, Fisheries and Food (MAPAQ) showed an annual incidence of injuries caused by domestic animals of 2.43 per 1000 people in northern Québec, compared to a rate of 0.1 to 0.7 per 1000 people in southern regions, with 60 to 96% of those injuries being related to dogs [13]. Such disparities have been mentioned in studies of other parts of Canada, the US and around the world, and they appear to be associated to inequalities in socioeconomic status (SES) [14–16]. In Alaska, there was a significantly higher mean annual rate of dog bites in Native people compared to non-Natives (0.1 versus 0.03/1000 inhabitants, respectively) [14].

Most studies have pointed out that dog bites are also an unequal burden when it comes to age, as children are more likely to be the victims of dog attacks due to their physical attributes and limited cognitive development, which leads to poor judgment in risky situations [4, 17–19]. A report published by the Canadian Hospital Injury Reporting and Prevention Program (CHIRPP) shows that dog bites feature among the five most common causes of injury in children aged from 5 to 9 y/o [20]. According to the same study, the hospitalization rate is almost three times higher for children aged 1 to 4 y/o compared to the general population (all ages).

Nunavik is a vast land area that covers one third of the province of Québec, Canada and has a very low population density [21]. Nunavik is inhabited by indigenous people, mainly Inuit, in 14 remote villages along the coasts of Hudson Bay and Ungava Bay [22]. Dogs play an important role in Innuit communities, impacting both the physical and mental health and well-being of humans. Through their participation in daily activities such as hunting, displacements and travel, companionship, protection and guarding, they have always been recognized as key members of the community [23–25]. This is despite various present-day problems that seem to be associated with roaming dogs and uncontrolled dog populations [26].

In Nunavik, Arctic fox rabies has been endemic since 1945 and constitutes a serious public health threat as evidenced by rabid animals being detected almost every year since then [4, 27, 28]. Although dogs are not a direct reservoir for the disease, they play an important role as an intermediary between wildlife and humans for rabies exposure, mainly through dog bites [6]. Despite the efforts of public health and public safety sectors, this issue remains unresolved [26]. In fact, little research has been conducted to explore all facets of the problem and identify sustainable solutions. Furthermore, even though multiple studies have investigated the epidemiology of dog bites, few if any have explored the specific and general context surrounding dog bites and their management from a one-health perspective [17, 29, 30].

The purpose of this study is to retrospectively describe human cases of potential exposure to rabies in Nunavik, particularly those involving dogs, and to determine if there are differences between children and adult cases. This study also sets the benchmark for establishing and assessing the Dog Program in Kuujjuaq, a program that was started in January 2020 and encompasses potential measures and interventions aimed at mitigating the risks for public health at the human-dog interface.

## Methods

This epidemiological study used a mixed design. It retrospectively analyzed an available database of human cases of potential exposure to rabies in 2008–2017. We also collected contextual information related to rabies dynamics, dog population and health, and the management and reporting of potential human exposure to rabies in order to ensure a more accurate and reliable interpretation of the results of the database analysis.

### Epidemiological analysis

#### Data sources

The tidied database of human cases of exposure to rabies as well as the dictionary of variables were provided by the Nunavik Regional Public Health Board (NRPHB). This database had been previously developed, updated and cleaned [31]. The information contained in this database is mainly derived from report forms filled out by frontline health professionals for cases of potential human exposure to a rabid animal [5]. The completed *management form* and the *rabies test report* provide data on the victim and the animal, respectively. We obtained demographic information on the Nunavik population over the study period from Québec’s Statistics Institute, and used this data for the standardization of incidence rates.

#### Variables

Th original database contained demographic information about the victim (sex, age in years, village where the exposure occurred), the date of the incident (exposure date), and the date of the reporting to health authorities (reporting date). When the exposure date was not given, the reporting date was used instead. Site of exposure, type of exposure and the animal involved were also available. Data on post-exposure prophylaxis (PEP) included the recommendation decision (PEP decision). Information on animal management included: follow-up information (if the animal was put under observation or analyzed for rabies), whether the animal was killed before the observation period was over (culled before) and, if the animal had been tested for rabies, the test results. Finally, a free text field for comments was available.

We created four age groups for descriptive analyses (1: 0–4 y/o; 2: 5–14 y/o; 3: 15–34 y/o; 4: 35+ y/o). These were restricted to two (children: 0–14; adults: 15+ y/o) for the multivariable analysis. For cases involving a dog, we used the content of the free text field to analyze the circumstances in which the injury occurred if the information provided was useful. We defined a priori three main categories of circumstances after a review of the relevant literature on aggressive behavior in dogs [5, 29, 32–35]. These were: 1) injuries occurring during play or as a form of communication, 2) injuries occurring as a dog reaction to a perceived threat (protection of food, litter or territory, fear of being harmed, or redirected aggression), and 3) injuries occurring as an intended aggression or predation by the dog. We assigned a category to each case based on a search for pre-defined keywords in the free text field. When information on the circumstances was ambiguous, we coded the circumstances as inconclusive. When the free text field did not include information about the circumstances, we coded the circumstances as not applicable (NA).

#### Availability of data

Information on the following variables was rarely absent (< 5%): age of the victim, sex, village, animal type, exposure date and reporting date. However, there was more missing data for other variables such as exposure type (16.2%), exposure site (39.4%), PEP (27.5%) and the free-text field for the cases where the animal involved was a dog (51.8%).

#### Statistical analysis

We used descriptive statistics to describe the cases. Mean annual incidence per 1000 inhabitants was calculated for each village and adjusted for age and sex based on 2017 Nunavik population data using direct standardization [36, 37]. Uniformity tests (two-sided Kolmogorov-Smirnov test) were performed to determine if exposures occurred uniformly across months and across weekdays.

Differences between age groups relative to the other variables were explored in three steps. We started with a bivariable description of age and each other variable, then a multivariable description, and ended with formal statistical analyses. The multivariable description was performed using a multiple correspondence analysis (MCA) that explored potential patterns between modalities of the following variables: sex, age group, exposure site, exposure type, PEP decision, animal type, animal follow-up and rabies test result. Univariable and multivariable analyses using binary logistic regression were conducted within two case scenarios (all exposures and exposures through dog only). The threshold for statistical significance was set at a *p*-value of 0.05. We performed all statistical analyses on R 3.4.2 software using the Stats package for the multivariable analysis, and the MASS package for the multiple correspondence analysis.

### Context analysis

#### Data collection

Using individual semi-structured interviews, we collected information on case management in northern Québec, as well as on changes and major events that might have occurred during the study period and across the 14 villages. We used a mixed approach for recruiting participants: purposive sampling followed by identifying key stakeholders involved directly or indirectly in animal and/or the human case management. Further participants were then recruited upon referral by initial interviewees. Human health professionals were asked for details on case management in Nunavik, while animal health specialists were asked about animal management and/or Arctic rabies dynamics during the study period. Local stakeholders were mainly asked about dog population dynamics and the current situation regarding dogs in Nunavik (e.g. use of restraints, vaccination and licensing). The interview guide detailed four to six open-ended questions, with the exact number of questions posed varying depending on the extent of the involvement of the participant and their organism in exposure case management. These questions were: 1) What are your organization’s roles and activities, and how do they affect case management or animal related injury risk in Nunavik? 2) What changes could have occurred during the study period (2008–2017) in those roles and activities? 3) What changes or major events could have occurred from month to month or season to season? 4) In your opinion, are there any differences between the 14 villages? 5) In your opinion, are there any differences between the age groups in regard to these roles and activities? 6) Does your organization have any documentation or additional data related to your answers? The interview guides were developed for the present study (see Additional files 1 to 4).

The consent form as well as the interview guide would be sent beforehand to the participants. Interviews were conducted in person whenever the research team could meet the participant, otherwise, it was conducted via the phone. Data collection started in November 2018 and ended in March 2019. Each interview was recorded after obtaining consent from the participants. Notes were also taken by the research team during interviews. The data were then stored for transcription and analysis.

#### Data analysis

After each interview, the recording and notes were used to write a brief summary, which was then sent to the participant for validation. The validated summaries were then manually triangulated and analyzed based on the interview questions.

Through interviews with stakeholders, we were able to collect additional information on domestic animal-related injuries in Québec for 2015 and 2016 from MAPAQ, and on rabies dynamics in Nunavik during the study period from the Canadian Food Inspection Agency (CFIA).

## Results

### Overall description of human cases of potential exposure to rabies

In total, 320 human cases of potential exposure to rabies were reported in Nunavik from 2008 to 2017. Of these, 293 (92%) involved a dog. Cases associated with wildlife were mainly linked to Arctic foxes (68% of 23 cases). Overall, the victims were more often men (62% of all cases) than women, the proportion being greater among cases not associated with dogs (87% men) (Table 1). Children aged 5 to 14 years accounted for one third of all cases (Table 1). The majority of injuries were located in the upper limbs (40%) or lower limbs (35%).

**Table 1:** Demographic characteristics of potential human exposures to rabies in Nunavik for 2008–2017

Variable	All injuries (*n* = 320)		Dogs (*n* = 293^a^)		Other animals (*n* = 23^a^)	
	n	%	n	%	n	%
Sex						
Male	198	61.9	176	60.1	20	87
Female	120	37.5	115	39.2	3	13
NA	2	0.6	2	0.7	0	0
Age group						
[0–4]	30	9.4	30	10.2	0	0
[5–14]	110	34.4	102	34.5	5	21.7
[15–34]	114	35.6	101	34.5	12	52.2
[35+]	61	19.1	55	18.8	6	26.1
NA	5	1.6	5	1.7	0	0

^a^ The total (dogs + other animals) is not 320 because animal type was unknown in 4 cases

**Fig. 1:**
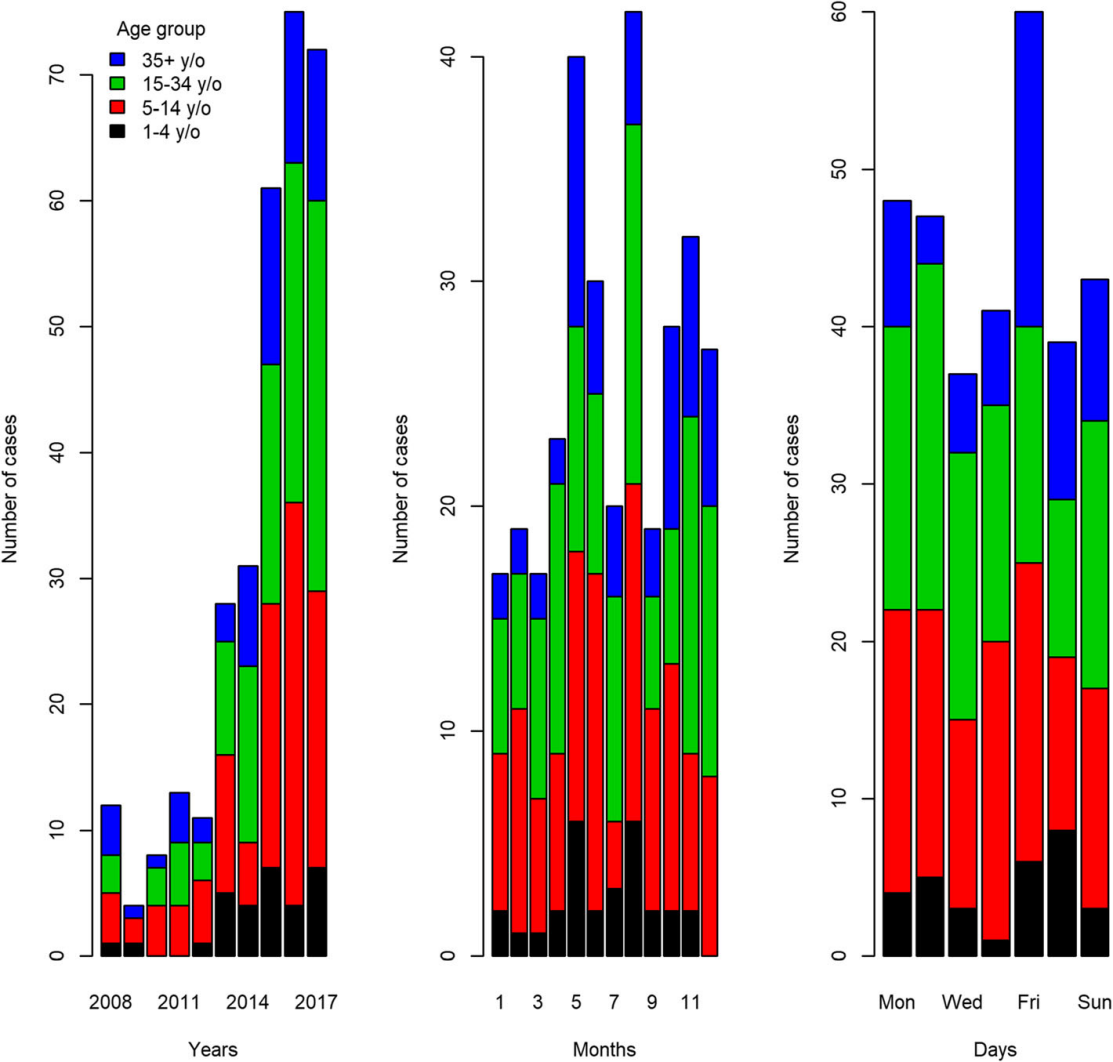
Distribution of cases of potential human exposures to rabies by years, months, and days of the week by age group, Nunavik 2008–2017

The incidence of potential rabies exposure reported to the public health authority increased remarkably during the study period: a two-fold increase from 2014 (*n* = 31) to 2017 (*n* = 72) (Fig. 1). Overall, the incidence was not uniformly distributed across months, nor across days of the week (K-S test: D = 1; critical value = 0.08) (*p*-value < 0.01), with May and August associated with 12 and 13% of cases, respectively, and Friday 18% (Fig. 1).

**Fig. 2:**
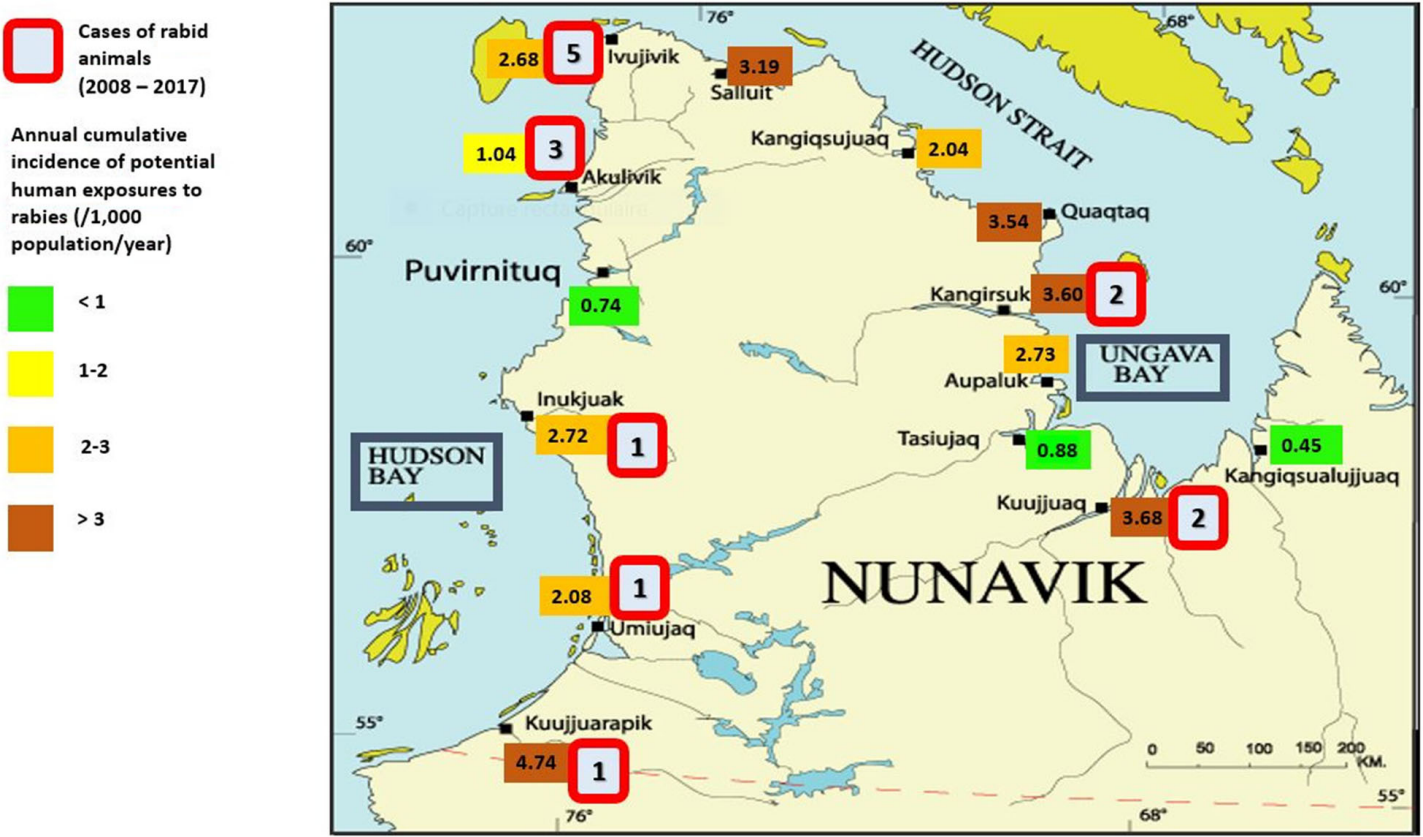
Adjusted annual cumulative incidence (/1000 population) of potential human exposures to rabies and number of positive rabid animal per village, Nunavik 2008–2017. Map source: Makivik Corporation. https://www.makivik.org/wp-content/uploads/2013/02/nunavik1.gif

The adjusted annual incidence by village over the study period varied between 0.45 and 4.6 cases per 1000 inhabitants in Kangiqsualujjuaq and Kuujjuarapik, respectively (Table 2). Visually, incidences appear to be higher in the villages of Ungava Bay compared to Hudson Bay (Fig. 2).

**Table 2:** Distribution of potential human exposures to rabies by village in Nunavik for 2008–2017

Village (sorted by decreasing adjusted annual incidence)	n	%	Mean annual incidence (per 1000 people)	Mean adjusted annual incidence (per 1000 people) ^a^
Kuujjuarapik	31	9.7	4.69	4.74
Kangirsuk	21	6.6	3.69	3.60
Quaqtaq	14	4.4	3.59	3.53
Kuujjuaq	88	27.5	3.50	3.68
Salluit	47	14.7	3.28	3.19
Inukjuak	48	15.0	2.76	2.72
Ivujivik	10	3.1	2.63	2.68
Umiujaq	10	3.1	2.19	2.08
Kangiqsujuaq	17	5.3	2.11	2.04
Aupaluk	4	1.2	2	2.73
Akulivik	7	2.2	1.09	1.04
Tasiujaq	3	0.9	0.95	0.88
Puvirnituq	14	4.4	0.81	0.74
Kangiqsualujjuaq	5	1.6	0.45	0.45
NA	1	0.3	–	–
Total	320	100	2.5	2.5

^a^ Cumulative incidences were standardized for both age and sex using direct standardization

PEP was recommended for 43% of the cases for which the information was available. It was more frequently recommended in cases involving wildlife (86%) compared to dogs (38%).

Sixty-two percent of dogs involved were held for observation following the exposure; a minor fraction (1.6%) of them were killed before the follow-up was over. Observation rate increased over the study period from 25% (3/12) in 2008, to 83% (60/72) in 2017.

Fifteen rabid animals (6.7% of all cases) were identified during the study period, of which 9 (60%) were dogs. Proportion of positive results was 4% among dogs, and 43% among wild animals. The number of rabid animals ranged from 0 to 2 per year, except for in 2015 and 2017, when it was 6 and 4, respectively. These animals were located in seven different villages with one, Ivujivik, accounting for 33% of all positive cases (Fig. 2).

PEP was recommended for all confirmed exposures with only one exception; in this instance, careful examination of the case file revealed that the person was in contact with an animal carcass and took all the necessary precautions to avoid contamination while handling it. Therefore, the assessment at the time concluded there were no risk of exposure to rabies. PEP recommendation was a posteriori not required in 35% of cases, where the animal involved was tested and found not rabid.

The free text field included information in 152 dog-related cases (51.8% of all cases). Around 40% of these cases were coded as a dog reaction to a human threat, 17% occurred during play, and 7% were coded as a direct aggression by the dog, whereas the information was not useful in 38%. Most exposures during play involved children up to 15 y/o. For reaction due to a perceived threat, around 63% of all cases were seen in victims aged 15 y/o and older. Cases where the aggression was intended “predation” were more or less equally distributed between the four age groups (Table 3). The free text field for the nine cases associated with a rabid dog provided no information about the bite circumstances.

**Table 3:** Characteristics of potential human exposures to rabies by age group and percentage of the total in each group, Nunavik 2008–2017

Variables	Age groups											
	Total		[0–4]		[5-14]		[15-34]		[35+]		NA	
	n	%	n	%	n	%	n	%	n	%	n	%
Sex												
Male	198	61.9	16	53.3	56	50.9	84	73.7	41	67.2	2	20.0
Female	120	37.5	14	46.7	54	49.1	30	26.3	20	32.8	1	40.0
NA	2	0.6	0	0	0	0	0	0	0	0	2	40.0
Exposure type												
Bite	244	76.2	26	86.6	81	73.6	87	76.3	46	75.4	4	80.0
Percutaneous	8	2.5	0	0	5	4.5	2	1.8	1	1.6	0	0
Mucous	16	5	2	6.7	4	3.6	8	7.0	2	3.3	0	0
NA	52	16.2	2	6.7	20	18.2	17	14.9	12	19.7	1	20.0
Exposure site												
Disseminated	15	4.7	2	6.7	4	3.6	3	2.6	6	9.8	0	0
Lower limbs	67	20.9	5	16.7	24	21.8	22	19.3	16	26.2	0	0
Upper limbs	77	24.1	6	20.0	24	21.8	36	31.6	11	18.0	0	0
Head and neck	32	10	7	23.3	15	13.6	8	7.0	2	3.3	0	0
Trunk	3	0.9	1	3.3	1	0.9	0	0.0	1	1.6	0	0
NA	126	39.4	9	30.0	42	38.2	45	39.5	25	41.0	5	100
Animal type												
Dog	293	91.6	30	100	102	92.7	101	88.6	55	90.2	5	100
Other	23	7.2	0	0	5	4.5	12	10.5	6	9.8	0	0
NA	4	1.2	0	0	3	2.7	1	0.9	0	0	0	0
PEP												
Recommended	99	30.9	7	23.3	27	24.5	34	29.8	28	45.9	3	60.0
Not recommended	133	41.6	16	53.3	41	37.3	56	49.1	20	32.8	0	0
NA	88	27.5	7	23.3	42	38.2	24	21.1	13	21.3	2	40.0
Exposure season												
Winter	65	20.3	3	10.0	25	22.7	24	21.1	11	18.0	2	40.0
Spring	80	25	9	30.0	25	22.7	30	26.3	16	26.2	0	0
Summer	93	29.1	11	36.7	33	30.0	34	29.8	14	23.0	1	20.0
Autumn	81	25.3	6	20.0	27	24.5	26	22.8	20	32.8	2	40.0
NA	1	0.3	1	3.3	0	0	0	0	0	0	0	0
Exposure day												
Weekday	237	74.1	19	63.3	85	77.3	87	76.3	42	68.9	4	80.0
Weekend	83	25.9	11	36.7	25	22.7	27	23.7	19	31.1	1	20.0
NA	0	0	0	0	0	0	0	0	0	0	0	0
Animal test result												
Positive	15	4.7	1	3.3	2	1.8	10	8.8	2	3.3	0	0
Negative	210	65.6	19	63.3	72	65.5	74	64.9	41	67.2	4	80
NA	89	27.8	10	33.3	33	30.0	28	24.6	17	27.9	1	20
N/App	6	1.9	0	0	3	2.7	2	1.8	1	1.6	0	0
Exposure circumstances^a^												
Play/ communication	26	17.1	7	43.8	12	21.8	5	10.2	1	3.6	1	25.0
Reaction	59	38.8	5	31.2	16	29.1	25	51.0	12	42.9	1	25.0
Aggression/ predation	10	6.6	2	12.5	3	5.5	1	2.0	4	14.3	0	0
Inconclusive	51	33.6	1	6.2	24	43.6	16	32.7	10	35.7	0	0
N/App	6	3.9	1	6.2	0	0	2	4.1	1	3.6	2	50.0

^a^Cases including dogs only (*N*= 152)

### Children versus adults

**Fig. 3:**
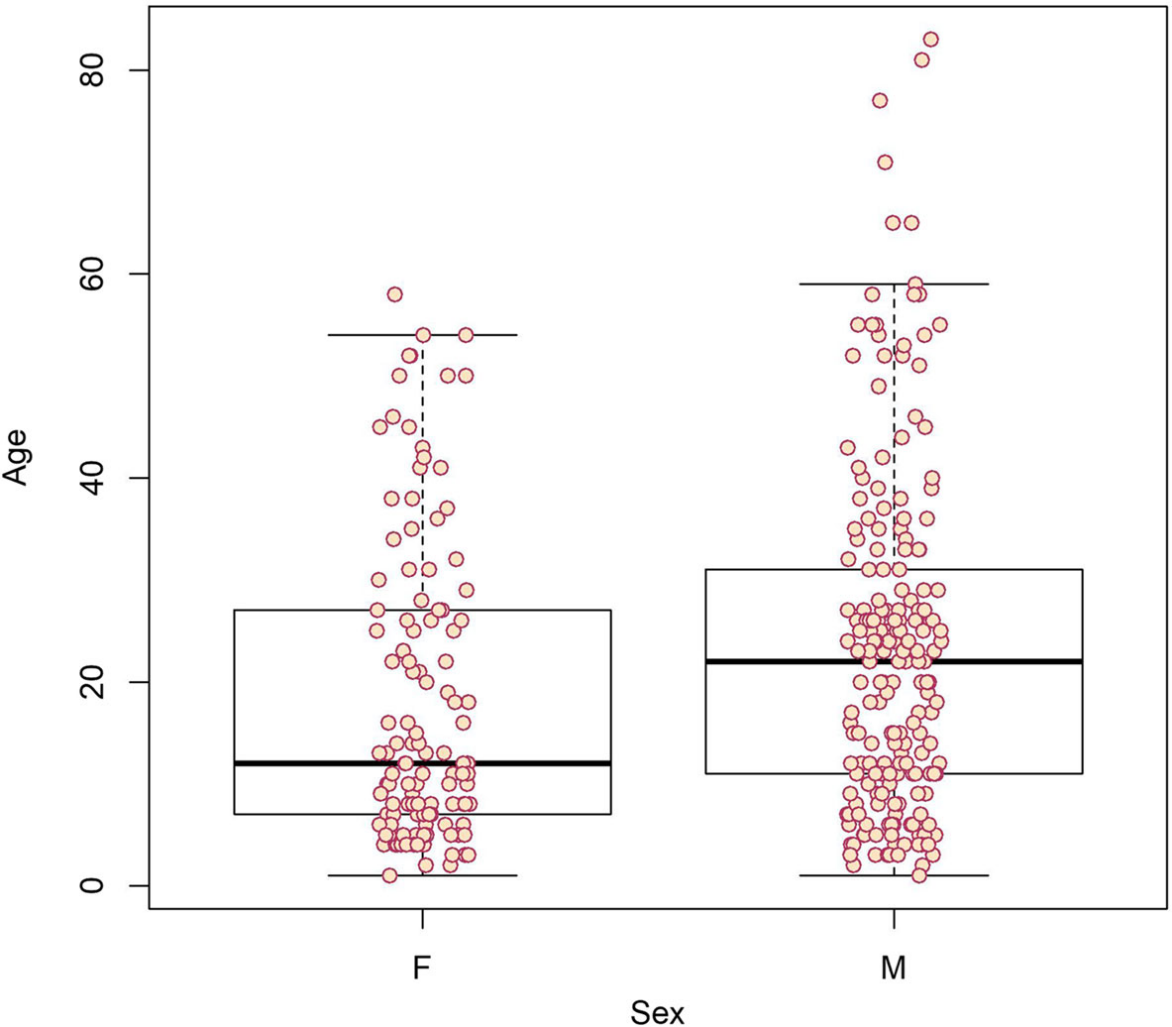
Age distribution of potential human exposures to rabies by sex, Nunavik 2008–2017

The bivariable description showed differences between age groups. Notably, the age distribution included a cluster of very young victims (below 10 y/o) in both sexes, and another cluster in the early 20s in males (Fig. 3). Accordingly, more males than females were observed in the two older age groups (Table 3). Very young children (0–4 y/o) were potentially exposed only through dogs and through bites (86.6%), the exposure site for them was more frequently the head and neck (23.3%), and they were less frequently injured during the winter compared to the other age groups. PEP was more recommended more frequently for the oldest age group (15+ y/o), and one-third (34%) of PEP were administered to victims aged 15 to 34 y/o.

**Fig. 4:**
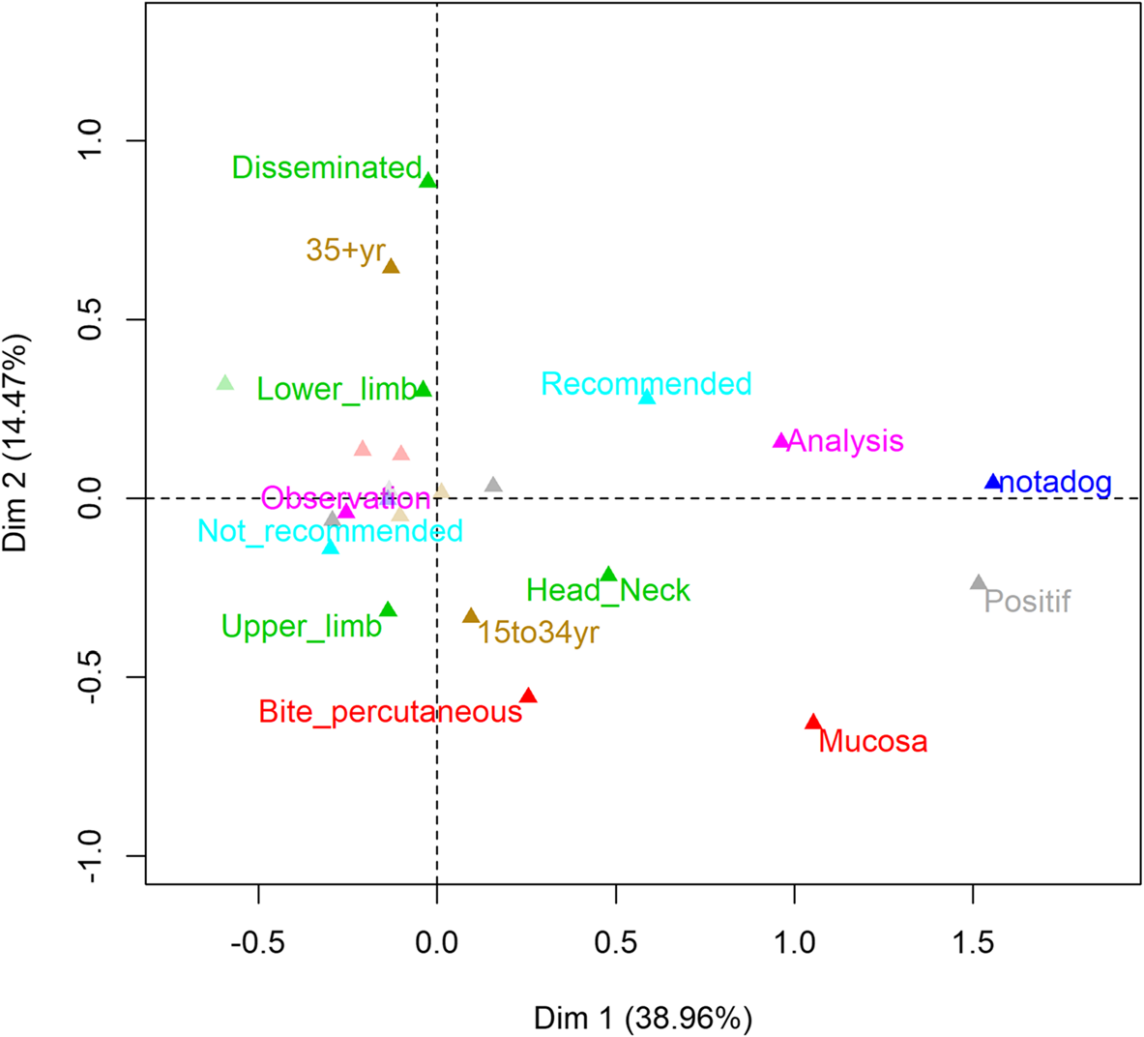
MCA plot (or projection) on the first two dimensions of age group (0–4, 5–14, 15–34, 35+ y/o; in orange), sex (M, F) and the following variables describing the exposure: the exposure site (lower limbs, upper limbs, head or neck, disseminated, trunk; in green), the exposure type (bite, mucosa, percutaneous, bite and percutaneous; in red), the animal involved (dog, not a dog; in blue), the PEP recommendation decision (recommended, not recommended; in light blue), the animal follow-up (observation, analysis; in pink) and the animal test result for rabies (negative, positive; in light gray)

MCA was conducted on the 86 cases for which information was available for all variables of interest. The first two dimensions accounted for 39 and 14% of total inertia (amount of variation in the data), respectively, and so these variables were used for interpretation. Overall, the figure shows a partial association along the first dimension between the following case features: animal not being a dog, positive rabies test result, analysis of the animal, exposure through mucosa, and recommendation for PEP. The second dimension features some differences between cases aged 15–34 y/o and those older. The latter were relatively more associated with disseminated injury or injury to the lower limbs and PEP recommendation, whereas cases of 15–34 y/o were relatively more associated with injury to the upper limbs, head or neck, percutaneous exposure and no PEP recommendation. No obvious association with age groups was found except for age group 4 (35+ y/o), which was associated with the second dimension, determined by exposure site (upper and lower limbs) and exposure type (bite and percutaneous) (Fig. 4).

Univariable logistic regression analysis showed that victims aged 0–14 y/o were more frequently females and were more likely to get injured by dogs compared to older victims (Table 4). No other variables were significantly associated with age group. No significant association was observed in the multivariable regression analysis of all types of exposure, whereas the head or neck as the exposure site was the only statistically significant result for dog-related exposures (Table 4).

**Table 4:** Logistic regression results using children as the reference group

Variable	Model a: All exposures				Model b: Exposures through dogs only			
	Univariable		Multivariable		Univariable		Multivariable	
	OR (95% CI)	*p-*value	OR (95% CI)	*p-*value	OR (95% CI)	*p-*value	OR (95% CI)	*p-*value
Sex								
Male	1.00	–	1.00	–	1.00	–	1.00	–
Female	2.38 (1.49–3.85)	0.0003	1.37 (0.74–2.50)	0.23	2.32 (1.43–3.85)	0.0006	1.54 (0.81–2.94)	0.19
Exposure site								
Disseminated	1.00	–	1.00	–	1.00	–	1.00	–
Head and neck	3.30 (0.94–12.42)	0.07	3.38 (0.95–12.85)	0.06	3.67 (0.99–14.63)	0.05	3.75 (1.00–15.06)	0.05
Upper limbs	0.96 (0.31–3.11)	0.94	0.97 (0.31–3.18)	0.95	0.90 (0.28–3.01)	0.86	0.85 (0.26–2.87)	0.79
Lower limbs	1.14 (0.37–3.76)	0.82	1.16 (0.37–3.84)	0.80	1.05 (0.32–3.55)	0.93	1.05 (0.32–3.57)	0.93
Torso	3.00 (0.23–73.58)	0.41	2.59 (0.20–64.12)	0.48	2.67 (0.21–65.79)	0.46	2.36 (0.18–58.85)	0.52
Animal								
Other	1.00	–	1.00	–	–	–	–	–
Dogs	3.05 (1.18–9.42)	0.03	2.22 (0.78–7.30)	0.15	–	–	–	–

### Context

Ten participants at the local, regional and provincial levels were interviewed: eight were animal or human health professionals, and two were locals.

The management and reporting process in Nunavik for a human case of potential exposure to rabies can be described as follows (at the time of the interviews). If a victim injured by an animal seeks medical attention in Nunavik, the case is usually reported to and treated by the local community service centre (CLSC). All of the 14 Nunavik villages have a CLSC. Frontline nurses are in charge of case management and follow-up, which is guided by a treatment algorithm. The case is immediately reported to NRPHB and to MAPAQ, which provides expertise on rabies exposure risk if the animal involved is domestic, or passes it to Québec’s Ministry of Forests, Wildlife and Parks (MFFP) if it is a wild animal. At the local level, animal control agents, in collaboration with MAPAQ or MFFP (as applicable), are in charge of follow-up if the animal is traceable, and for preparing and sending the carcass to the CFIA rabies reference laboratory for testing.

The interviewees identified two major events related to case management processes. First, around 2014, the CFIA, which had previously been involved in rabies surveillance in animals, saw its role significantly reduced to just rabies diagnostic testing. Other activities, including carcass or head preparation and expedition, were transferred to other provincial and regional authorities, which led to more responsibilities for NRPHB and MAPAQ. Second, at the local level, health professionals also noted that a remarkable change in the processes and tools available for case management and reporting between 2015 and 2016. In brief, the documents that frontline health professionals relied on were modified and made available online to facilitate the decision-making process in case management.

Respondents did not mention differences across months or seasons specific to their roles and responsibilities. However, they stated that, in general, cases tended to be more frequent during summer. Some linked the dog-related injuries and potential exposure to rabies to activities such as boating and fishing, during which people often leave their dogs to roam. It also appears that dog population dynamics are affected by seasonal outbreaks of highly contagious infectious diseases such as parvovirus and distemper. From an exclusively wild animal perspective, participants pointed out that most cases of exposure involving fauna, and almost all rabies cases in wild animals, are seen during the cold season (October to March), due to activities such as hunting for fur-bearing animals or the scarcity of food resources in wild habitats during this season. These variations were further confirmed by the rabies test results in animals provided by the CFIA: more than 69% of the rabid animals observed during the study period were tested between December and March.

Interviewees pointed out some issues with animal control services; for example, positions tend to be very instable, leading to a lack of continuity at the local level. Moreover, a lack of education and awareness often leads to a lack of cooperation from locals, whether for dog control efforts, PEP or follow-up after an injury. Furthermore, participants said the effectiveness of such control requires greater involvement and commitment from officers.

Participants confirmed that there are no differences regarding their roles and activities in managing exposure cases associated with age. However, healthcare professionals were unanimous on the disproportionate over-representation of children compared to adults and the severity of the wounds seen in children under 5 y/o. Most respondents related these differences to the inappropriate behavior of children and adolescents toward dogs in Nunavik. As for adults, participants mentioned that the activities of hunting and mushing are risk factors, especially for males.

## Discussion

The purpose of this study was to characterize cases of potential human exposure to rabies in Nunavik, determine differences in this exposure between children and adults, and interpret the findings contextually. We estimated the mean annual incidence of potential human exposure to rabies in Nunavik at 2.5 cases per 1000 people. We identified 15 exposures with actual rabid animals and highlighted the significance of dogs in cases of potential and actual exposure of humans to rabies: 92% of exposures were associated with dog bites, and 9 of the 15 rapid animals were dogs. These actual exposures and the incidence of potential exposure to rabies in Nunavik is almost 10 times higher than what has been previously reported in Canada [7, 20], confirming that rabies and potential exposure to rabies is still a significant public health concern in this region. It is worth noting that no cases of human rabies have occurred in Nunavik thanks to the strict case management of all potential rabies exposures by medical authorities. This study reveals that potential cases were all managed adequately, regardless of the age and gender of victim involved, time of exposure, and Nunavik village involved.

### Dog bites and human exposure to rabies in Nunavik

The figures for the 14 remote Inuit villages in Nunavik confirm the higher risk of dog bites and potential exposure to rabies reported in other remote and indigenous communities in North America [14, 38–40]. This higher risk has been linked to socioeconomic status and other determinants such as structural, social or cultural disparities, including limited access to animal health services and animal training capacities, along with insufficient law enforcement and education on responsible pet ownership [16, 41, 42]. In addition, the abundance of stray or free-roaming dogs have been blamed for the high incidence of dog bites in remote indigenous communities [26, 43–45]. This suggests that reducing potential exposure to rabies through dog bites requires a range of measures applied to specific modifiable determinants, such as law enforcement, education (of children, parents, dog owners, etc.) and dog health services. Inducing change in some determinants may take much longer, in particular socioeconomic and cultural ones.

### Exploring patterns of potential human exposure to rabies

Our study highlights two major profiles of victims based on age, sex and the animal involved. The first profile is children aged up to 14 y/o, male or female, who are mostly exposed through dogs when playing with them. They were also more likely to sustain injuries in the head and neck. This seems universal to children and is no doubt related to their particular physical and developmental characteristics [30, 46, 47].

The second profile is young adult males (aged 15 to 34 y/o), who are exposed through wildlife or dogs (following a reaction type of aggression) and who sustain injuries to the upper limbs. In addition, exposure during outdoor activities such as hunting or mushing occur mainly to this profile. Men in northern communities traditionally practice such activities during fall or winter. This association with age and gender has been mentioned in other studies [17, 48, 49]; however, the pattern seems to vary depending on social environment and related risk factors, as illustrated in our study.

### Exposure circumstances

Our investigation of exposure circumstances in cases involving dogs confirms that most injuries (56%) were the result of an intentional or unintentional form of “provocation”. Although the use of the term “provoked” is very controversial, it has been acknowledged that some forms of interactions are highly prone to elicit aggressive behavior in dogs linked to territoriality, protection and guarding, or fear [5, 33, 47, 50]. Although sometimes labeled as “play-bites” or positive interactions [51], it is important to distinguish situations where the human and the dog are playing from those where the intention to play comes only from the victim’s side [34]. Misinterpretations of a dog’s signaling behavior and an inappropriate attitude on the part of the human in a risky situation (e.g. female with litter, a sleeping dog) are often overlooked, especially in the case of children, despite the fact that they play a major role in triggering aggressive behavior [51–53].

### Temporal distribution of reported cases over the study period

Reported cases of potential exposure to rabies in Nunavik increased significantly from 2013 to 2017. Our contextual exploration, which even included a specific question about temporal trends, failed to provide a clear explanation for this rise in cases. We have evidence that rabies in wildlife was rather stable over the study period and that dog populations did not increase; hence, the hypotheses that there were more frequent contacts between human beings and dogs or rabid animals is not supported. Two significant temporal changes, however, were reported: a redistribution of responsibilities in activities associated with rabies surveillance in animals in 2014–2015, and improvements to the processes and tools available for the management and reporting of potential human exposure to rabies for frontline medical and public health staff. It is difficult to see a link between the first change and the increased number of potential exposures to rabies. The second change might have led to increased reporting; however, considering that rabies has been a public health issue for a long time in the region, it is difficult to believe that frontline staff were not already diligent in the management and reporting of potential exposure prior to these changes. A more probable explanation might be that changes within the medical and public health system indirectly raised awareness about rabies and the importance of medical consultation in the event of exposure among the general public, leading to an increase in the number of people consulting medical services for potential exposure to rabies.

No clear pattern of seasonality was found, although our results show that more cases of injuries were declared during the months of May and August, which is similar to previous findings [48, 54]. One hypothesis is that contact with dogs increases during the long photoperiod season, especially since dogs are usually kept outside in Nunavik [44]. In addition, the context analysis revealed that during seasonal activities such as boating and fishing, locals tend to stay away from their homes for days, leaving their dogs roaming unleashed.

### Policy implications

Although cases of rabid animals were rare and more or less stable over the ten-year period, potential exposures appeared to be higher in villages on the Hudson Bay compared to Ungava Bay. Information related to rabies dynamics in wildlife, as well as the epidemiological factors contributing to the contact rate between wild and domestic animals, is needed to identify the hot spots and design effective prevention programs [4, 55]. Mass vaccination of dogs can also be a barrier for human exposure; however, since the immunization status of dogs is not well documented in Nunavik, it is difficult to estimate coverage rates and their impact. This highlights the importance of law enforcement in the context of an endemic setting.

The findings regarding victim profiles demonstrate the need to tailor specific prevention programs. Furthermore, future prevention programs should focus on aspects such as educating people, especially children, on dog body language and appropriate/safe interactions.

### Study strengths and limitations

A clear contribution of this study comes from its use of data for both human and animal variables that were systematically collected and compiled over a long and recent period. The second strength was our thorough uni- and multivariable description of all data available prior to the formal testing for differences in potential exposure to rabies associated with age. The last major strength is the context analysis used to help interpret our findings.

As a retrospective study, some biases are inherent to the collection and analysis of data; for example, by using only registries of reported cases rather than actively collecting the information, we might have overlooked a fraction of actual potential exposures with possibly different characteristics [8, 56]. Considering that no human rabies cases have occurred in the region for several decades, we can be relatively sure that we were able to assess genuine exposure to rabies, meaning that our figures on incidence may underestimate the risk for potential exposure but not the risk of exposure to rabid animals. Missing data for some variables tended to limit the validity of estimates and their use for statistical analysis; nevertheless, missing data were rare for the most important variables, especially those involved in defining the two victim profiles. Obviously, the study targets Nunavik and so its findings cannot be generalized to other parts of the Arctic without caution.

## Conclusion

This study quantifies the risk of potential exposure to rabies and the relative contribution of dog bites to this risk in northern Inuit villages in Québec. Although rabies is a real public health concern in Nunavik, medical services and their partners have been diligent in managing all cases of potential rabies exposure, including close follow-up or testing of the animal involved. This has resulted in no actual cases of human rabies.

We identified and characterized two specific risk profiles for potential exposure to rabies in Nunavik based on age and gender: children (female or male) bitten by a dog while playing with it, and young male adults in contact with wildlife during outdoor activities. Both profiles deserve targeted prevention programs to reduce the burden of potential rabies exposure.

This study shows an increase in reported cases of potential exposure to rabies. We could find no obvious explanation for this phenomenon, although we hypothesize that it is due to an increased awareness in the general public about rabies, which may be an indirect effect of process changes in management and reporting within the health sector. This increase in incidence needs to be confirmed for the years after 2017. We also need to investigate the reasons for such an increase, especially considering the possible impacts on human life (e.g. a possible increase in true exposure to rabies) and on the health system (e.g. more cases to manage and to report, more PEPs and follow-ups).

## Supplementary information

**Additional file 1.** Interview guide for Public Health professionals

**Additional file 2.** Interview guide for representatives of the Ministry of Agriculture, Fisheries and Food of Québec

**Additional file 3.** Interview grid for representatives of the Quebec Ministry of Forests, Wildlife and Parks

**Additional file 4.** Interview guide for the representatives of the municipalities of Nunavik

## Data Availability

The data that support the findings of this study are kept by the Public Health Department of Nunavik but due to restrictions on their availability they are not publicly available. However, as we used the data under license for the current study, we can provide the data upon reasonable request and with permission from the Public Health Department of Nunavik. For the context analysis, data can be obtained upon reasonable request from the corresponding author: Sarah Mediouni (sarah.mediouni@umontreal.ca).

## Abbreviations

CFIA: Canadian Food Inspection Agency
CHIRPP: Canadian Hospitals Injury Reporting and Prevention Program
CLSC: Local Community Services Centers
MAPAQ: Ministry of Agriculture, Fisheries and Food of Québec
MCA: Multiple correspondence analysis
MFFP: Ministry of Forests, Wildlife and Parks of Québec
NRPHB: Nunavik Regional Public Health Board
PEP: Post-Exposure Prophylaxis
SES: Socioeconomic status
