# Supplementary material for: Epidemiology of human exposure to rabies in Nunavik: incidence, the role of dog bites and their context, and victim profiles
Source: BMC Public Health. 2020 Apr 29;20:584. doi: 10.1186/s12889-020-08606-8 (PMC7191815; doi:10.1186/s12889-020-08606-8)
Supplement: Supplementary file 4 — Additional file 4. Interview guide for the representatives of the municipalities of Nunavik [file 12889_2020_8606_MOESM4_ESM.docx]

Thank you for participating in this study. We will begin the interview with a general question about your organization before asking 5 more specific questions

You work for …………………………………..

**Question 1** : Can you explain to me the roles and activities of your organization that can directly or indirectly influence the bite risk in Nunavik, for example, the municipal by-laws on dogs, their application (dog pound, slaughtering), the vaccination of dogs against rabies.

**Question 2**: Here's a timeline:

2008 2009 2010 2011 2012 2013 2014 2015 2016 2017

What do you think has changed or may have changed in these roles and activities between 2008 and 2017?

**Question 3** : Here are the months of a year:

1 2 3 4 5 6 7 8 9 10 11 12

What do you think is changing or could change over the course of a year in these roles and activities?

**Question 4** : Here is a map of Nunavik (source: Kativik Regional Government):


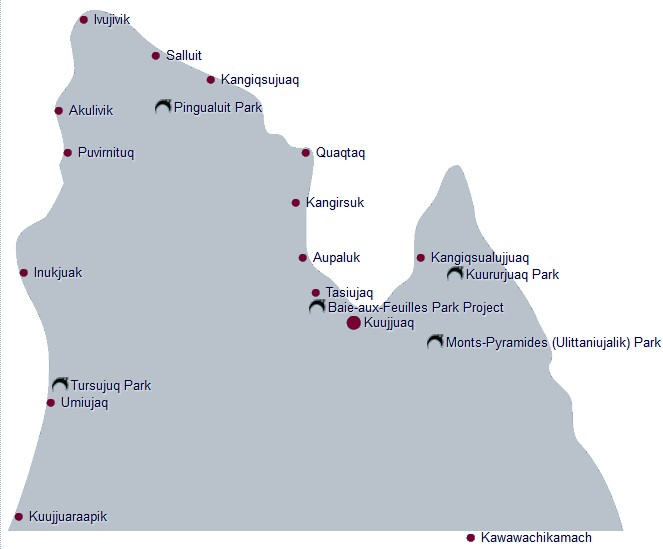


What do you think is different or could be different between the villages in Nunavik or between the east (Ungava Bay) and west (Hudson Bay) coasts in these roles and activities?

**Question 5** : Not applicable

**Question 6** : According to you, does your organization have any documents or data that would detail the answers you have just provided? If so, what are these documents or data and who can we contact to see if we can access them?

That was our last question. We're going to make a written summary of what you said and then send it to you so that

you can comment on it and modify it before it becomes final. How can we send you the abstract?

By email ..............................................................................................................................................................................................

Thank you again for your participation and have a good end of the day!
